# A Backward Walking Training Program to Improve Balance and Mobility in Children with Cerebral Palsy

**DOI:** 10.3390/healthcare9091191

**Published:** 2021-09-09

**Authors:** Ji-Young Choi, Sung-Min Son, Se-Hee Park

**Affiliations:** 1 Department of Physical Therapy, College of Health Science, Cheongju University, Cheongju 28503, Korea; saviorglory@hanmail.net; 2 Department of Physical Therapy, Graduate School of Health Science, Cheongju University, Cheongju 28503, Korea; sehui7688@naver.com

**Keywords:** backward walking, balance, cerebral palsy

## Abstract

Background: We studied the effects of motor tasks using backward walking training on balance and gait functions of children with cerebral palsy. This was a single-blinded, randomized controlled trial with a crossover design conducted at a single facility. Methods: Among 12 children with cerebral palsy, the forward (FWG) (*n* = 6) and backward walking groups (BWG) (*n* = 6) underwent training three times a week for 4 weeks, 40 min a day. After a 6-week break, the crossover training was conducted. Functional walking variables were measured. Time-Up-and-Go (TUG) test, Figure-8 Walk Test (FW8T), and Pediatric Balance Scale (PBS) were used for measuring balance. Results: Both groups showed significant improvement in walking speed, stride length, and step length. The BWG demonstrated significant improvement in walking speed (*p* < 0.05) compared with the FWG. The TUG test, FW8T, and PBS showed significant improvement. After the 4-week intervention, both groups displayed a remarkable decrease in TUG duration and FW8T. Both groups also exhibited improvement in the PBS; more so in the BWG. Conclusions: Backward walking training with motor dual tasks could be a more effective interventional approach than forward walking training to improve balance and walking functions of children with spastic hemiplegia.

## 1. Introduction

Cerebral palsy is a non-progressive lesion that is described as a group of permanent disabilities of motor performance caused by injuries in the brain of a developing fetus or infant [1]. Cerebral palsy is accompanied by disturbances in language, the sensory system, and cognitive behavior, or epilepsy. There could be secondary musculoskeletal problems due to muscle weakness, which is attributed to malalignment, limited range of motion, and asymmetric posture [2]. These deformities are negatively influenced by biomechanical movement and can interfere with balance and gait function [3]. 

The independent gait in children with cerebral palsy has a great effect on the quality of daily life [4]. The major goal of rehabilitation in children with cerebral palsy is the recovery of independent gait. However, children with cerebral palsy have a restricted capacity for movement that results in gait dysfunction (a short stride, slow walking speed, increased swing phase, and postural instability). Thus, it is important to choose an effective training method to improve the balance and gait of children with cerebral palsy. Walking training may be efficient to improve both muscle tone, postural control, and gait function as well as muscle strength, endurance, and coordination of the lower extremity [5,6]. Particularly, dual-task training, which refers to the capacity to control two tasks simultaneously, can be a very effective way to improve the balance and gait function of children with cerebral palsy [7,8].

Walking while performing activities of daily living (ADL) involves the use of inter-joint coordination and the performance of cognitive processes, such as moving things, manipulation of tools, and talking to others [9]. Thus, dual-task training that combines walking while performing other tasks is required. Stroke patients underwent dual-task training while walking to improve their balance and walking, and dual-task training affects their daily life and balancing ability [10]. Chronic stroke patients who lacked confidence improved in their dual-task performance based on walking training, which had a positive effect on their gait and self-efficacy [11]. Dual-task training enhanced ADL and balance in children with ataxic cerebral palsy. They were trained using ring hanging, cup stacking, and blocks and performed motor dual tasks to enhance weight shifting while catching toys in various directions [12]. Therefore, children with cerebral palsy need an intervention program consisting of walking and dual-task training for performing meaningful movements. 

Forward-walking training (FWT) is usually conducted, but backward-walking training (BWT) is a more effective intervention in postural conditioning and walking. BWT improves postural balance and walking ability in children with cerebral palsy [13] and was more significantly associated with the speed and step length of stroke patients’ than FWT [14]. Based on previous studies, BWT may lead to better functional improvement than FWT. However, several studies have been focused on the positive effects of BWT in stroke patients, but none were related to children with cerebral palsy. Moreover, no studies have investigated the effects of dual-task training while walking backward.

Therefore, this study aimed to identify the effectiveness of BWT combined with motor tasks in children with spastic hemiplegic cerebral palsy. Our objective included determining the effects of this training on balance and gait function in children with spastic hemiplegic cerebral palsy, which was the novelty of this study.

## 2. Materials and Methods

### 2.1. Participants

This study was a randomized controlled trial with a crossover design conducted at a single rehabilitation facility. Twelve children (aged 7–14 years) with a diagnosis of spastic hemiplegic cerebral palsy participated in this study. The inclusion criteria were as follows: (1) children with Level I or II cerebral palsy classified according to the Gross Motor Function Classification System (GMFCS) who could move independently without an assistive device; (2) children with a score of 15 or higher in the Cognitive Assessment of Functional Independent Measure (WeeFIM); and (3) those who did not participate in a BWT or a specific training before 6 months. 

The study was approved by the Institutional Review Board of Cheong-ju University, and parents of all children provided signed informed consent to participate in the study. The general characteristics of the participants are described in Table 1.

### 2.2. Procedures and Intervention

**Figure 1:**
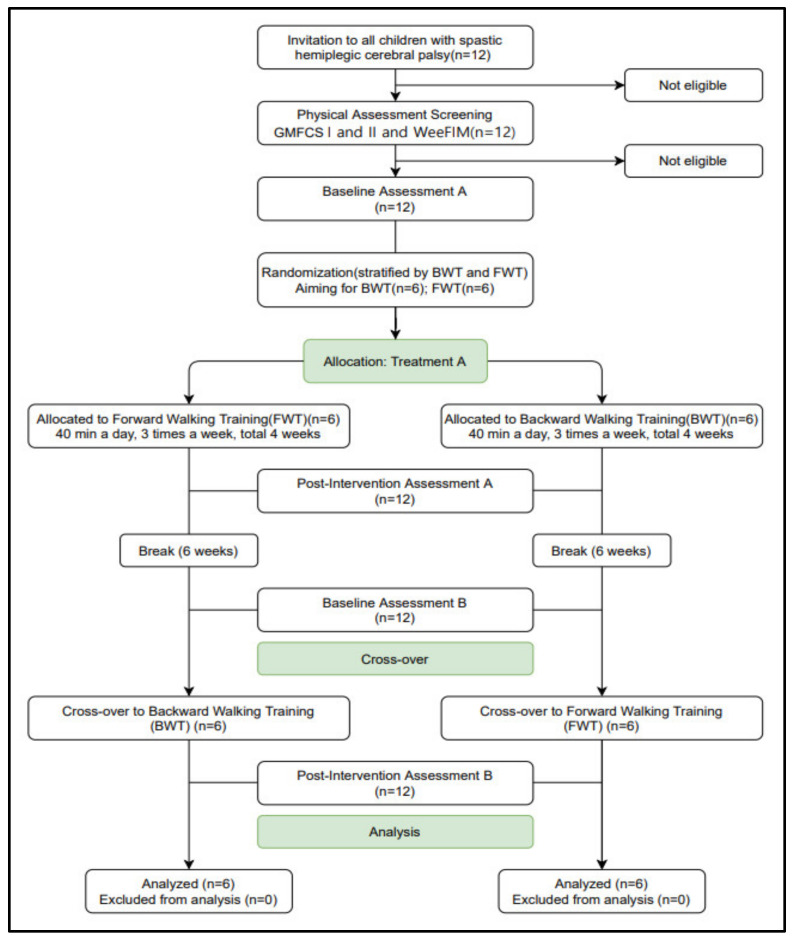
Flowchart in this study. Assessment A, B: Time Up and Go, Figure-8 Walk Test, Pediatric Balance Scale, Opto gait.

This study used a two-group randomized trial crossover design, and the children were randomly divided into the FWT and the BWT groups. Both groups received 4 weeks of BWT and 4 weeks of FWT (the training order of one group was FWT and BWT, and in the other group was BWT and FWT), with a washout period of 6 weeks during the post-intervention phase of the 1st period and the pre-intervention phase for the 2nd period. The assessments were measured at four time points, that is, during the pre- and post-intervention phases of the 1st and 2nd periods. Of the 12 participants recruited initially, none dropped out during the intervention period (4 weeks), and all 12 subjects (FWT, *n* = 6; BWT, *n* = 6) were included in the final analysis. The experimental design of this study is provided in Figure 1.

#### 2.2.1. Backward Walking Training (BWT)

BWT included gait training on the ground without obstacles. Before BWT, general physical therapy (such as stretching, range of motion exercises, and strengthening exercises) was performed for 10 min. Three sets of BWT with motor dual tasks were executed as per the following schedule: 7 min per set, with approximately 3 min of inter-set rest, for a total training duration of 30 min. The method of BWT applied was based on that described by Davies [15]. First, the therapist helped the participants to move the lower limb correctly when walking backward. The therapist gradually decreased the assistance when the participants underwent training. Second, the participants were then trained to cover a distance of approximately 15 m along the corridor of the treatment room using a safety bar held by the hand on the unaffected side. Third, the participants were encouraged to walk independently without a safety bar. Finally, the participants attempted to walk at a comfortable pace of their choice and gradually increased the distance and speed during backward walking. The training was facilitated by a physical therapist with more than 5 years of experience. 

#### 2.2.2. Forward Walking Training (FWT) 

During FWT, the therapist assisted in correcting the position of the participant’s foot to accurately complete the different elements of the gait cycle. The walking velocity was determined by the pace of the participant. FWT also included the same components, such as general physical therapy and task performance, as BWT.

#### 2.2.3. Motor Task

During the BWT and FWT, the participants were asked to carry a plastic cup containing water (height = 15 cm, base diameter = 6 cm) on a tray. The task was to transport the plastic cup filled with water (using both hands) without spilling its contents, and dropping the cup was considered a failure [14].

### 2.3. Measurement

#### 2.3.1. Evaluation for Participant Selection

(1) Cross Motor Functional Classification System (GMFCS)
  GMFCS is a classification system used for measuring the level of motor disorders in children with cerebral palsy. It is divided into five stages according to movements, such as sitting, crawling, and walking, and the degree of mobility using assistive equipment. From level I, which includes children who can walk without any limitations in movement, to level V, which includes difficulty in movements even with assistive equipment; the higher the step, the lower the functional mobility. The inter- and intra-rater reliabilities are 0.93 and 0.97–0.99, respectively [16].
(2) Pediatric Functional Independent Measure for Children (WeeFIM)
  WeeFIM is a tool used to assess a child’s functional ability based on their health, development, education, and social conditions. It is divided into areas of motor and cognitive function, classified into six lower measures, and evaluated using 18 items. Among them, communication, and social cognition are evaluated under the cognitive function. Communication is evaluated for possible comprehension and expression and social interactions. Problem-solving skills and memory are evaluated under social cognition. The evaluation method is conducted by direct observation and interviews by the therapist, and each item is scored on a 7-point scale from 1 to 7. The validity and inter-rater reliability are excellent (intraclass correlation coefficients > 0.90) [17,18].

#### 2.3.2. Outcome Measures

(1) Time-Up-and Go Test
  TUG is a test that can quickly measure dynamic balance and functional mobility over time. It comprises measuring the time taken from getting up from an armchair, walking 3 m, turning around, and walking back 3 m to sit in place. The participant sits with his/her feet flat on the floor so that the hips and knees are in 90° flexion. The therapist measures the time taken to walk 3 m three times and records the average value. It takes about 11 to 12 s for disabled participants, and if it takes more than 20 s, it is determined that help is needed when walking. For the TUG test, the intra- and interrater reliabilities are ICC = 0.99 and ICC = 0.99, respectively. It is a very reliable measurement method [19].
(2) Figure-8 Walk Test (FW8T)
  FW8T is a test performed to identify the ability to walk in different paths (straight, curved, clockwise, and counterclockwise) and to recognize the task. The FW8T requires the participant to walk a figure-8 around two cones placed 1.5 m apart. The therapist measures the time taken till the return and the step count. The participant is allowed to practice twice along the path of walking before measurement. The FW8T has excellent test–retest (ICCs = 0.84 and 0.82 for time and steps) and inter-rater reliability (ICCs = 0.90 and 0.92 for time and steps) [20].
(3) Pediatric Balance Scale (PBS)
  The PBS is developed for school-age children with mild and moderate motor disorders. The PBS assesses the balance and functional ADL and is available for use in children from 5 to 15 years old. It consists of 14 items (with five grade levels) including sitting balance, standing balance, sit-to-stand, stand-to-sit, moving from chair to chair, standing on one leg, rotating 360 degrees, reaching to the floor, and reaching forward turning. The performance of each task is evaluated on a scale of 0 to 4 points. The PBS score is calculated as static (6 items), dynamic (8 items), and total components (total score), with a maximum total score of 56. The higher the score, the better the balance [21]. The PBS has shown excellent intra-class correlation coefficient (ICC > 0.9) and inter-rater reliability (ICC > 0.9) [22].
(4) Opto Gait
  We used the Opto Gait system (OPTOGait, Microgate, Bolzano, Italy, 2010) consisting of three transmitting and three receiving bars, to collect data on the participants’ walking characteristics. Two bars are placed parallel to each other 1 m apart. Ninety-six LED diodes are positioned on each bar 1 cm apart, 3 mm above the ground. When the participant passes between the transmitting and receiving bars, the system detects the interruption of the optical signal and automatically calculates the spatiotemporal gait parameters based on the presence of a foot in the recording area. The first Opto Gait bar is placed 50 cm from the starting point. The spatiotemporal gait parameters, such as the speed, stride length, and step length of the affected side were analyzed.

### 2.4. Statistical Analysis

The results of both the interventions, including the 1st and 2nd periods, were pooled. The data were analyzed using SPSS version 22.0 for Windows (IBM, Armonk, NY, USA). To identify the statistically significant differences between pre- and post-test values in the gait and balance function variables (Opto Gait, TUG, FW8T, and PBS) of the BWT and FWT groups, Wilcoxon signed-rank tests were used. The Mann–Whitney U test was used for inter-group comparisons. The statistical significance level was set at 0.05.

## 3. Results

Twelve participants with spastic CP (5 males, 7 females; mean ± standard deviation age: 10 ± 2.48 years; height = 125 ± 9.99 cm; weight = 27.33 ± 7.35 kg) were included in this study. Participants were either in the GMFCS level I (*n* = 8) or II (*n* = 4) (Table 1).

The TUG test scores decreased from 13.85 ± 2.00 and 14.90 ± 1.73 to 13.24 ± 2.24 and 13.33 ± 1.70 in the FWT and the BWT groups, respectively. There were statistically significant differences between the pre-test and post-test assessments in both groups (*p* < 0.05). The BWT group showed a greater reduction than the FWT group, and there was a significant difference in inter-group comparison between the two groups (*p* < 0.05).

In the FW8T, both groups revealed pertinent reductions in the post-test compared to the pre-test evaluations (*p* < 0.05). The difference between the pretest and post-test estimates in the BWT and FWT groups were −1.60 ± 0.48 and −0.70 ± 0.47, respectively. There was a significant difference in inter-group comparison between the two groups (*p* < 0.05).

Both groups displayed a significant increase in the intra-group comparison of PBS (*p* < 0.05). The difference between the pretest and post-test analyses in the BWT and FWT groups were 3.17 ± 0.40 and 1.33 ± 0.51, respectively. There was a significant difference in inter-group comparison between the two groups (*p* < 0.05) (Table 2).

There was a remarkable increase in the intra-group comparison of the spatiotemporal gait parameters (velocity, step, and stride) of both groups (*p* < 0.05). The differences between pre-test and post-test values in the BWT and FWT groups were 0.19 ± 0.11 and 0.06 ± 0.02, respectively. This suggests that only the walking speed increased significantly among the various spatiotemporal gait parameters. However, there were no significant differences in step and stride length (*p* > 0.05) (Table 3).

## 4. Discussion

This study aimed to investigate the effects of backward or forward walking combined with task-oriented training on balance ability (TUG, FW8T, and PBS) and functional gait in children with spastic hemiplegic cerebral palsy. The BWT and FWT groups showed significant differences in balance ability and spatiotemporal gait parameters after intervention. In the inter group comparison, the BWT group showed a more significant improvement in balance ability and walking speed parameters than the FWT group. The results of our study showed that backward walking with task-oriented training improved the balancing abilities and functional gait of children with spastic hemiplegic cerebral palsy, more so than those observed in the FWT.

The balancing ability or self-confidence required to improve the gait was higher during BWT because more sensory information is used for balance with limited visual information [23]. In addition, there is higher muscle activity and energy consumption than in FWT [24,25,26]. Moriello et al. [27] explained that they performed FWT and BWT for patients with spinal cord injury and that the balance and walking confidence improved significantly in the BWT group compared to the FWT group. The improvement in the experimental group is similar to the findings of El-Basatiny et al. [13], who reported that combining the use of BWT and traditional physical therapy in children with hemiparetic cerebral palsy resulted in better improvement in postural balance. Balance and walking combine information from the vestibular, visual, and proprioceptive senses to recognize the body spatially while being involved in posture control [28]. Therefore, backward walking stimulates proprioception, thereby facilitating the activation of righting and equilibrium reactions necessary for maintaining and adjusting posture. All these systems may be associated with the improvement of balance by BWT with dual motor tasks.

In our study, the BWT group showed an enhanced walking speed compared to the FWT group. The increased walking speed in the BWT group may prove the effectiveness of BWT in improving gait capacity. Several mechanisms have been suggested to explain the benefits of BWT in children with spastic cerebral palsy. First, BWT might help increase muscle strength more than FWT. Devita et al. [29] reported that BWT showed a significant increment of muscle activation (quadriceps, hamstring, and tibialis anterior muscle) in stroke patients. An approximate 20% increase in the quadriceps muscle strength was noted during its measurement using a dynamometer. Compared to FWT, BWT resulted in a more eccentric contraction in the quadriceps muscle. Eccentric contraction is more efficient in enhancing muscle strength due to higher energy consumption than concentric contraction [30]. Backward walking induces greater joint movement and ground reaction forces and results in greater energy consumption [31]. Additionally, the interaction of the quadriceps of the knee and flexor digitorum longus of the ankle joint plays an important role in propulsion during the stance phase of backward walking [32]. The momentum gained by the propulsion of the quadriceps during the stance phase might contribute to the enhancement of muscle strength [33]. Second, the BWT influences proprioception, which can contribute to the improvement of gait function. Proprioception is an essential element to maintain balance and serves to control movement when walking [24,25,26]. Backward walking due to limited vision utilizes proprioception more for the balance response. Although initial motor learning required a high dependence on visual information, proprioceptive information had a greater effect in the learned motor control stage [34]. Mullie et al. [35] reported that stroke patients use their ankle proprioception when walking with their eyes closed. In addition, BWT can induce greater activation of the cerebrum than FWT. 

Peters et al. [36] also suggested the backward walking increases the activity of both supplementary and primary motor area of the cerebral cortex compared to forward walking. An analysis of brain activation during backward walking showed that hemodynamic cortical activations in the motor area were greater during backward walking than during forward walking [37]. The improvement in walking speed of children with cerebral palsy could be due to muscle strengthening and activation of proprioceptive information. Therefore, the present study showed a greater positive influence due to BWT with dual motor tasks than due to FWT in children with spastic hemiplegic cerebral palsy.

The limitations of this study are as follows. First, it is difficult to generalize the results for all children with cerebral palsy due to the small number of participants. Second, our study only included children with spastic hemiplegic cerebral palsy with GMFCS levels I and II. Our findings demonstrate that balance function in children with cerebral palsy could be positively influenced by BWT with motor dual task training rather than FWT. Therefore, to redress these limitations, future studies should investigate the results of our study in children with various types of cerebral palsy and use longer washout periods with larger sample sizes. 

## 5. Conclusions

This study aimed to investigate the effect of BWT combined with motor task on balance function and walking capacity of children with CP. Our results demonstrate that children with spastic hemiplegic CP exhibit greater improvements in the balance function (TUG, FW8T, PBS) and walking capacity (gait velocity) following BWT combined with motor tasks than following FWT combined with motor tasks. Our findings suggest effective intervention to promote the ability to balance to prevent fall injuries through BWT and promote self-care activities in daily life. Future studies with larger sample sizes and longer washout periods should explore the effects of BWT and FWT in children with other types of CP as well.

## Figures and Tables

**Table 1:** General characteristics of the subjects (N = 12).

**Variables**	**M ± SD**
Sex (male/female)	5/7
Affected side (Rt./Lt.)	7/5
GMFCS level (Ⅰ/Ⅱ)	8/4
Age (years)	10 ± 2.48
Height (cm)	125 ± 9.99
Weight (kg)	27.33 ± 7.35

M ± SD: mean ± standard deviation. Rt.: Right side, Lt.: Left side. GMFCS: Gross Motor Function Classification System.

**Table 2:** Comparison of changes in balance function in between groups.

		Forward (n = 12)	Backward (n = 12)	U	*p* ^(2)^
		M ± SD	M ± SD		
TUG	Pre	13.85 ± 2.00	14.90 ± 1.73		
	Post	13.24 ± 2.24	13.33 ± 1.70		
	Post-pre	−0.60 ± 0.59	−1.57 ± 0.31	4.50	0.03
	*p* ^(1)^	0.02	0.02		
FW8T	Pre	12.20 ± 3.57	12.73 ± 2.74		
	Post	11.50 ± 3.31	11.12 ± 2.45		
	Post-pre	−0.70 ± 0.47	−1.60 ± 0.48	2.00	0.01
	*p* ^(1)^	0.02	0.02		
PBS	Pre	42.17 ± 5.03	41.33 ± 4.67		
	Post	43.50 ± 4.88	44.50 ± 4.63		
	Post-pre	1.33 ± 0.51	3.17 ± 0.40	0.00	0.00
	*p* ^(1)^	0.02	0.02		

TUG: Time-Up-and-Go test, FW8T: Figure-8 Walk Test, PBS: Pediatric Balance Scale. ^(1)^ Wilcoxon Rank-sum test, ^(2)^ Mann–Whitney test.

**Table 3:** Comparison of changes in gait function in between groups.

		Forward (n = 12)	Backward (n = 12)	U	*p* ^(2)^
		M ± SD	M ± SD		
Velocity (m/s)	Pre	0.69 ± 0.09	0.66 ± 0.11		
	Post	0.75 ± 0.09	0.85 ± 0.14		
	Post-pre	0.06 ± 0.02	0.19 ± 0.11	2.00	0.00
	*p* ^(1)^	0.00	0.00		
Step (cm)	Pre	39.46 ± 4.27	38.54 ± 4.10		
	Post	43.29 ± 2.57	43.91 ± 3.62		
	Post-pre	3.83 ± 3.51	5.37 ± 3.55	50.50	0.21
	*p* ^(1)^	0.00	0.00		
Stride (cm)	Pre	85.38 ± 3.09	87.62 ± 4.55		
	Post	80.50 ± 5.30	80.50 ± 3.42		
	Post-pre	4.87 ± 4.97	7.12 ± 4.83	50.50	0.21
	*p* ^(1)^	0.00	0.00		

^(1)^ Wilcoxon Rank-sum test, ^(2)^ Mann–Whitney test.

## Data Availability

All of the relevant data are presented within the manuscript. All data are public.
